# The *PIT1 *gene polymorphisms were associated with chicken growth traits

**DOI:** 10.1186/1471-2156-9-20

**Published:** 2008-02-27

**Authors:** Qinghua Nie, Meixia Fang, Liang Xie, Min Zhou, Zhangmin Liang, Ziping Luo, Guohuang Wang, Wensen Bi, Canjian Liang, Wei Zhang, Xiquan Zhang

**Affiliations:** 1 Department of Animal Genetics, Breeding and Reproduction, College of Animal Science, South China Agricultural University, Guangzhou 510642, Guangdong, China

## Abstract

**Background:**

With crucial roles on the differentiation of anterior pituitary and the regulation of the prolactin (*PRL*), growth hormone (*GH*) and thyroid-stimulating hormone-β (*TSH-β*) genes, the chicken *PIT1 *gene is regarded as a key candidate gene for production traits. In this study, five reported polymorphisms (MR1-MR5) of the *PIT1 *gene were genotyped in a full sib F_2 _resource population to evaluate their effects on growth, carcass and fatty traits in chickens.

**Results:**

Marker-trait association analyses showed that, MR1 was significantly associated with shank diameters (SD) at 84 days (P < 0.05), hatch weight (HW) and shank length (SL) at 84 days (P < 0.01), MR2 was significantly associated with BW at 28, 42 days and average daily gain (ADG) at 0–4 weeks (P < 0.05), and MR3 was significantly associated with ADG at 4–8 weeks (P < 0.05). MR4 was associated with SL at 63, 77, 84 days and BW at 84 days (P < 0.05), as well as SD at 77 days (P < 0.01). Significant association was also found of MR5 with BW at 21, 35 days and SD at 63 days (P < 0.05), BW at 28 days and ADG at 0–4 weeks (P < 0.01). Both T allele of MR4 and C allele of MR5 were advantageous for chicken growth. The *PIT1 *haplotypes were significantly associated with HW (P = 0.0252), BW at 28 days (P = 0.0390) and SD at 56 days (P = 0.0400). No significant association of single SNP and haplotypes with chicken carcass and fatty traits was found (P > 0.05).

**Conclusion:**

Our study found that polymorphisms of *PIT1 *gene and their haplotypes were associated with chicken growth traits and not with carcass and fatty traits.

## Background

As a kind of POU (Pit-Oct-Unc)-domain binding factor, pituitary-specific transcription factor (PIT1, or GHF1, or POU1F1) has been proved to bind and transactivate promoters of growth hormone (*GH*), prolactin (*PRL*) and thyroid-stimulating hormone-β (*TSH-β*) genes [1-3]. Other bioactivities of PIT1 have also been reported, like regulating anterior pituitary development [4,5] and pituitary cell proliferation [6], silencing or delaying adrenarche in human [7], being related to dwarf phenotype in mice [8], as well as inducing the differentiation of hepatic progenitor cells into PRL-producing cells [9]. Initially activated under the control of *Phophet of PIT1 *(*PROP-1*) gene, *PIT1 *gene is auto-regulated in expression [10] and its mRNA is present in any cell types of pituitary, whereas PIT1 protein mainly expresses in lactotrophs, somatotrophs and thyrotrophs, which secrete PRL, GH and TSH-β [11].

Until now, *PIT1 *cDNA has been identified in a variety of species, and previous studies showed that the *PIT1 *gene comprised 6 exons in mammals and 7 exons in birds and fishes, seen as differences in precursor length [12-14]. The chicken *PIT1 *cDNA has firstly been isolated and sequenced by Tanaka et al. (1999) [15], and its three isoforms of PIT1*, PIT1β* and PIT1ω* induced by alternative splicing have also been isolated and found to comprise 335, 363, and 327 amino acids, respectively [16]. The alternative splicing of *PIT1 *gene has also been reported in other species [17,18]. According to the chicken genome sequences released in May of 2006 [19], the chicken *PIT1 *gene is located at chromosome 1 (GGA1) and spans over 14 kb in length.

Due to its crucial regulatory function and a variety of bioactivities, *PIT1 *has been regarded as a key candidate gene for production performance. There are indications that variations of *PIT1 *gene are related to growth, carcass and fatty traits in pig [20-24], growth and carcass traits in cattle [25,26]. In chickens, although a total of 23 single nucleotide polymorphism (SNP) and a 57 bp indel have been lately identified in 2400 bp discrete region of *PIT1 *gene, their genetic effects on chicken production traits remain unclear [27]. Recently, it has been shown that a non-synonymous SNP at POU domain (A → T, Asn229Ile) is significantly associated with body weight at 8 wk [28].

In this study, five reported polymorphisms of the chicken *PIT1 *gene were genotyped in a full sib F_2 _resource population to evaluate their genetic association with chicken growth, carcass and fatty traits were also observed.

## Methods

### Chicken populations

A full sib F_2 _resource population as described by Lei et al. (2005) was used in this study [29]. The cross of 9 White Recessive Rock (WRR) males and 9 Chinese Xinghua (X) females and the reciprocal cross of 6 WRR females and 6 X males produced 17 F_1 _families and 454 F_2 _full-sib individuals. F_2 _chickens were raised in floor pens and fed with commercial corn-soybean-based diets that met all NRC requirements. All birds from three generations were genotyped to evaluate the effects of *PIT1 *variations on chicken production performance. All 57 tested traits were 31 growth traits of HW, BW at 7, 14, 21, 28, 35, 42, 49, 56, 63, 70, 77, 84 and 90 days, SL at 42, 49, 56, 63, 70, 77, 84 and 90 days, SD at 42, 49, 56, 63, 70, 77 and 84 days, ADG at 0–4 and 4–8 weeks, and 14 carcass traits of head width (HWD), breast width (BWD), body length (BL), breast angle (BA), carcass weight (CW), eviscerated weight with giblet (EWG), eviscerated weight (EW), breast muscle weight (BMW), leg muscle weight (LMW), wing weight (WW), head and neck weight (HNW), shank and toe weight (STW), heart-liver- proventriculus-gizzard weight (HLPGW) and small intestine length (SIL), as well as 12 fatty traits of subcutaneous fat thickness (SFT), cingulated fat width (CFW), abdominal fat pad weight (AFW), leg muscle colour (LMC), breast muscle colour (BMC), shear force of leg muscle (SFLM), shear force of breast muscle (SFBM), leg muscle conductivity (LMCD), breast muscle conductivity (BMCD), water loss rate of muscle (WLRM), transversal area of the leg muscle fibre (TALMF) and transversal area of the breast muscle fibre (TABMF) [29].

### Markers and primers

Five reported polymorphisms of the chicken *PIT1 *gene that could be easily genotyped by either PCR-RFLP or simple PCR were selected as markers to evaluate their effects on chicken production traits. These polymorphisms were a 57 bp indel (MR1) [27] and 4 SNP (MR2-MR5). Four primer pairs of PR1-PR4 were designed and synthesized to amplify specific fragments covering MR1-MR5 (Table 1). The detailed information for these polymorphisms is presented in Table 1.

**Table 1:** Detail information for MR1-MR5 of the chicken *PIT1 *gene

Markers	Source^1^	Variation	Region	Primers (forward/reverse) (5' → 3')	Size (bp)^2^	Enzyme
MR1	Nie et al. 2005	57 bp indel	Intron 2	PR1 (gtcaaggcaaatattctgtacc/tgcatgttaatttggctctg)	387 or 330	/
MR2	Rs13905611	C/T	Intron 5	PR2 (ggaccctctctaacagctctc/gggaagaatacagggaaagg)	599	*TaqI*
MR3	Rs13687125	A/G	Intron 5	PR2 (as described above)	599	*MspI*
MR4	Rs13687127	C/T	Intron 5	PR3 (ggggattttgccactttaggg/tgggtaaggctctggcactgt)	442	*EcoRI*
MR5	Rs13687128	C/T	Exon 6 (I syn)	PR4 (tgggaagaacagtttatggc/tggctagcttgtaagggaatc)	483	*TasI*

^1 ^MR1 was reported by Nie et al. (2005); MR2-MR5 were released by NCBI with accession number of Rs13905611, Rs13687125 Rs13687127, and Rs13687128, respectively. The chromosomal sites for MR1-MR5 were nt 96213503–96213559, nt 96221521, nt 96221553, nt 96222619, and nt 96224228, respectively. ^2 ^indicated length of PCR products

### Genotyping by PCR-RFLP procedure

PCR was performed in 25 μL mixture containing 50 ng of chicken genomic DNA, 1 × PCR buffer, 12.5 pmol of primers (PR1-PR4), 100 μM of each dNTP, 1.5 mM MgCl_2 _and 1.0 U Taq DNA polymerase (Sangon Biological Engineering Technology Company, Shanghai, China). PCR was run in a Mastercycler gradient (M. J. Research Co Ltd, USA) with the following procedure: 3 min at 94°C, followed by 35 cycles of 30 s at 94°C, 45 s at annealing temperature (58–62°C), 1 min at 72°C, and a final extension of 5 min at 72°C. Genotypes of MR1 were directly observed by 2% agarose gel electrophoresis with PCR product amplified by PR1. PCR products of PR2-PR4 were further digested at 37°C overnight with *TaqI*, *MspI*, *EcoRI *and *TasI *(Table 1). The digestion mixture contained 8 μL PCR products, 1 × digestion buffer, and 3.0 U of each enzyme. Genotypes of MR2-MR5 were finally determined in TFM-40 Ultraviolet Transilluminator (UVP Company, USA) after 2.0% agarose gel electrophoresis of digestion mixture for half an hour.

### Statistical analyses

#### Haplotype inference

For 454 F_2 _individuals, genotype data of MR1-MR5 were used to infer haplotypes with PHASE 2.0 software [30].

#### Marker-trait association analyses

Marker-trait association analyses were performed with SAS GLM procedure (SAS Institute, 1996) and the genetic effects were analyzed using the following mixed model:

*Y = μ+G+D+H+S+e*

where Y is a trait observation, μ is the overall population mean, G is the fixed effect of genotype, D is the random effect of dam, H is the fixed effect of hatch, S is the fixed effect of sex (male or female), and e is the residual random error. With the above model, association of each of MR1-MR5 and their haplotypes with the 57 production traits was performed to evaluate its genetic effect on chicken growth, body composition and fat deposition.

## Results

### Genotype and haplotype inference

For each of MR1-MR5, three genotypes were found in the total population. Haplotypes inferred from genotype data showed that a total of 13 haplotypes were found. These haplotype contained two major ones of H1 ("DTGCC", 74.56% or 671 of 900) and H2 ("ITGTT", 12.89% or 116 of 900), six minor ones of H3 ('ITACC', 2.66% or 24 of 900), H4 ('ITGCC', 2.33% or 21 of 900), H5 ('ICGCC', 2.33% or 21 of 900), H6 ('ITGTC', 1.88% or 17 of 900), H7 ('DTGTC', 1.00% or 9 of 900) and H7 ('DCGCC', 1.00% or 9 of 900) with frequencies between 1% and 5%, as well as five rare ones (H9-H13) with frequencies lower than 1%.

### Association of single SNP with chicken production traits

Results showed that MR1 was significantly associated with SD at 84 days (P < 0.05) and highly significantly associated with HW and SL at 84 days (P < 0.01), and MR2 was significantly associated with BW at 28, 42 days and ADG at 0–4 weeks (P < 0.05). MR3 was significantly associated with ADG at 4–8 weeks (P < 0.05). Moreover, MR4 was significantly associated with SL at 63, 77, 84 days and BW at 84 days (P < 0.05) and highly significantly associated with SD at 77 days (P < 0.01), and T rather than C was advantageous for chicken growth (Table 2). MR5 was significantly associated with BW at 21, 35 days, and SD at 63 days (P < 0.05) and highly significantly associated with BW at 28 days and ADG at 0–4 weeks (P < 0.01), and C allele was advantageous for chicken growth (Table 3).

**Table 2:** Association of MR4 with chicken growth traits

Traits^1^	P value	Least squares Mean ± S.D^2^		
		
		CC (335)	CT (105)	TT (11)
**SL at 63 days (mm)**	0.0197	9.43 ± 0.14^ab^	8.99 ± 0.22^a^	10.00 ± 0.43^b^
**SL at 77 days (mm)**	0.0333	88.56 ± 0.82^a^	87.74 ± 1.34^a^	94.22 ± 2.59^b^
**SD at 77 days (mm)**	0.0065	9.90 ± 0.13^A^	9.61 ± 0.21^A^	10.81 ± 0.40^B^
**BW at 84 days (g)**	0.0207	1463 ± 19.36^a^	1436 ± 32.92^a^	1667 ± 82.23^b^
**SL at 84 days (mm)**	0.0346	88.28 ± 0.44^a^	87.41 ± 0.74^a^	92.13 ± 1.85^b^

^1^SL = shank length; SD = shank diameter; BW = body weight. ^2 ^Numbers in bracket referred to sample size for each genotype. ^a,b ^Means within a row with no common superscript differ significantly (P < 0.05). ^A,B ^Means within a row with no common superscript differ highly significantly (P < 0.01).

**Table 3:** Associations of MR5 with chicken growth traits

Traits^1^	P value	Least squares Mean ± S.E^2^		
		
		CC (331)	CT (92)	TT (28)
**BW at 21 days (g)**	0.0469	211.8 ± 1.92^a^	206.4 ± 3.67^ab^	196.4 ± 5.83^b^
**BW at 28 days (g)**	0.0045	313.3 ± 3.04^A^	301.8 ± 5.83^A^	281.0 ± 9.11^B^
**BW at 35 days (g)**	0.0153	441.3 ± 4.73^a^	415.6 ± 9.11^b^	402.5 ± 14.24^b^
**SD at 63 days (mm)**	0.0284	9.45 ± 0.13^ab^	8.93 ± 0.23^a^	9.69 ± 0.36^b^
**ADG at 0–4 weeks (g/d)**	0.0051	10.11 ± 0.11^A^	9.74 ± 0.21^A^	8.96 ± 0.32^B^

^1^BW = body weight; SD = shank diameter at 63 d of age; ADG = average daily gain. ^2 ^Numbers in bracket referred to sample size for each genotype. ^a,b ^Means within a row with no common superscript differ significantly (P < 0.05). ^A,B ^Means within a row with no common superscript differ highly significantly (P < 0.01).

None of these polymorphisms was significantly associated with any of chicken carcass and fatty traits (P > 0.05).

### Association of *PIT1 *haplotypes with chicken production traits

As far as haplotypes were concerned, a total of 428 individuals with 10 diplotypes (271 of H1H1, 62 of H1H2, 24 of H1H3, 15 of H1H6, 13 of H1H4, 10 of H1H5, 9 of H2H5, 8 of H2H4, 8 of H2H6, 8 of H2H7) were used in association analysis. Results showed that MR1-MR5 haplotypes were significantly associated with growth traits of HW (P = 0.0252), BW at 28 days (P = 0.0390) and SD at 56 days (P = 0.0400). Among ten diplotypes, H2H4 had much higher value of HW (mean = 30.2), BW at 28 days (mean = 352.6) and SD at 56 days (mean = 9.24) compared with other ones. Nevertheless, the *PIT1 *haplotypes were not significantly associated with any of chicken carcass and fatty traits (P > 0.05).

It was concluded that polymorphisms of *PIT1 *gene were associated with chicken growth traits, but not with carcass and fatty traits.

## Discussion and Conclusion

In this study, polymorphisms of the *PIT1 *gene were related to chicken growth traits. Until now, associations of the *PIT1 *gene with growth traits were reported in human [31], pig [19-23] and cattle [25,26]. In chicken, a non-synonymous SNP (Asn299Ile) in exon 6 of the *PIT1 *gene was significantly associated with body weight at 8 wk, and its allele frequencies differed significantly between meat-type and lay-type chickens [28]. Another SNP in exon 6 (MR5) was associated with ADG at 0–4 weeks, BW at 21, 28, 35 days and SD at 63 days as indicated by this study. Furthermore, three adjacent SNP in intron 5 (MR2-MR4) were associated with ADG at 0–4 and 4–8 weeks, BW at 28, 42 and 84 days, SL at 63, 77, and 84 days, as well as SD at 77 days. Until now, some QTL for body weight were identified in GGA1 [32-35], however, only one reported QTL covered the 96 Mb region (total chromosomal size of 201 Mb) where the chicken *PIT1 *gene located [36]. It seemed that these SNP may be in linkage disequilibrium with causative mutation(s), which situated in this region and played crucial roles on chicken growth.

It was interesting that the *PIT1 *gene polymorphisms were associated with none of chicken carcass and fatty traits. In previous studies, variations of the *PIT1 *gene were related to carcass and fatty traits in pig [20-22,24], carcass traits in cattle [25]. However, no association was found of five polymorphisms in the *PIT1 *gene with 26 carcass and fatty traits in this study. In addition, haplotype analysis also provided similar results. This was surprising because some carcass traits were correlated with growth traits to some extent, and therefore it still required further study for confirmation.

It was further indicated that polymorphisms of the *PIT1 *gene affected chicken growth at different stages. MR2, MR3 and MR5 seemed to have higher effects on chicken early growth, as they were associated with ADG at 0–4 and 4–8 weeks, BW at 21, 28, 35 and 42 days, and SD at 63 days, respectively. Otherwise, MR1 and MR4 seemed to have higher effects on chicken growth in middle stage, as they were associated with SL at 63, 77 and 84 days, SD at 77 and 84 days, and BW at 84 days. As far as different genotypes were compared, both T allele of MR4 and C allele of MR5 were advantageous for chicken growth.

It was concluded that polymorphisms of *PIT1 *gene and their haplotypes were associated with chicken growth traits.

## Authors' contributions

QN analyzed the data and drafted the manuscript. MF, XL and MZ participated in the data analyses. Both of ZL, GW, WB, CL and WZ carried out the genotyping studies. QN and XZ conceived of the study, and participated in its design and coordination. All authors read and approved the final manuscript.
